# Comparison of Side Effects of Nalbuphine and Morphine in the Treatment of Pain in children with Cancer: A Prospective Study

**DOI:** 10.3390/cancers14153617

**Published:** 2022-07-25

**Authors:** Anna Kubica-Cielińska, Michał Czapla, Raúl Juárez-Vela, Clara Isabel Tejada-Garrido, Marzena Zielińska

**Affiliations:** 1 Department and Clinic of Anaesthesiology and Intensive Therapy, Faculty of Medicine, Wroclaw Medical University, 50-556 Wroclaw, Poland; akubica@usk.wroc.pl (A.K.-C.); marzena.zielinska@umw.edu.pl (M.Z.); 2 Department of Paediatric Anaesthesiology and Intensive Care, University Hospital, 50-556 Wroclaw, Poland; 3 Department of Emergency Medical Service, Wroclaw Medical University, 51-516 Wroclaw, Poland; 4 Institute of Heart Diseases, University Hospital, 50-566 Wroclaw, Poland; 5 Group of Research in Care GRUPAC, Department of Nursing, University of La Rioja, 26004 Logroño, Spain; raul.juarez@unirioja.es (R.J.-V.); clara-isabel.tejada@unirioja.es (C.I.T.-G.)

**Keywords:** nalbuphine, morphine, pain treatment, children, cancer, opioid side effects

## Abstract

**Simple Summary:**

In the 2012 WHO guidelines for the treatment of chronic pain in children, the recommendation for the use of the weak opioids tramadol and codeine was removed, and in 2017 the indication for the use of these drugs for the treatment of postoperative pain in the paediatric population was also withdrawn. For the treatment of pain associated with cancer treatment, the recommendations of non-opioid analgesics and, in cases of insufficient analgesia, the use of strong opioids remained. Nalbuphine, a weak opioid commonly used in the treatment of perioperative pain in children, can also be used effectively in the treatment of cancer-related pain. Our study is the first to evaluate the quality of analgesia, side effects, and withdrawal syndrome in children receiving nalbuphine for the treatment of cancer pain. The results indicate that nalbuphine is an effective and safe analgesic that is less likely than morphine to cause adverse side effects when used to treat cancer-related pain in children.

**Abstract:**

Contemporary pain management regimens in children do not include the use of the middle step of the analgesic ladder, i.e., weak opioids. The aim of this study was to analyse the comparison of side effects and the therapeutic efficacy of morphine and nalbuphine in pain management in children with cancer. We conducted an observational, prospective study and analysed medical records of patients treated at the Clinic of Paediatric Haematology and Oncology of the University Hospital in Wroclaw (Poland), who developed mucositis during treatment. The efficacy and safety of both drugs were analysed, and the efficacy of pain relief and the incidence of adverse effects characteristic of opioid drugs were compared. The cases of 96 of children treated with opioid drugs nalbuphine or morphine were analysed. Nalbuphine therapy was accompanied by a statistically significantly lower incidence of side effects such as skin pruritus, constipation, and micturition disorders compared to morphine (*p* < 0.05). After the discontinuation of nalbuphine, signs of withdrawal syndrome were much less frequent than after morphine (*p* < 0.05). In Conclusion, nalbuphine used as a pain killer in children with oncological disorder is a safe drug. It provides stable analgesia in most children. Compared to morphine, the side effects typical of opioid use are less common, and the incidence decreases over time.

## 1. Introduction

Pain is inherent in oncological disease in children and there are many causes of pain in patients undergoing oncological disease treatment. The components of suffering include the pain resulting from the process of tumour growth and tissue damage, neuropathic pain resulting from damage to the nervous system, and iatrogenic pain accompanying the treatment process itself [1,2]. Paediatric oncological disease pain management is based on guidelines developed in 2012 by the World Health Organization (WHO) [3]. They recommend the use of a two-stage analgesic ladder. In the first stage, in the therapy of mild pain, acetaminophen and ibuprofen are recommended; in case of lack of effectiveness of such treatment, it is recommended to implement therapy with strong opioids. The primary opioid recommended for the treatment of severe pain is morphine. It is an effective and well-tolerated drug with high pain-relieving capacity, but not without side effects. The most common side effects of morphine accompanying its use are nausea, vomiting, dry mouth, and constipation, which cause considerable discomfort. Other common complications include difficulty urinating, bradycardia, and hypotension, as well as pruritus, which can be extremely distressing for the patient and is not always easy to treat. Despite these side effects, morphine remains the primary standard of care for chronic pain in children and the ‘gold standard’ for the use of opioids in the treatment of severe acute pain.

In addition to morphine, other potent drugs such as fentanyl, oxycodone, methadone, and buprenorphine are recommended for the treatment of chronic pain in children. Contemporary pain management regimens in children do not provide for the use of the middle step of the analgesic ladder, i.e., weak opioids. This creates a gap in the therapeutic regimen, which can to some extent be filled by nalbuphine, classified as a weak opioid.

Nalbuphine is a synthetic opioid of the phenanthrene derivative group, similar to the opioid antagonist naloxone and the agonist oxymorphone in its chemical structure. It exhibits receptor agonistic and antagonistic effects—agonistic for the ĸ receptor and antagonistic for the µ receptor. It is used to treat postoperative pain but also for the management of acute pain in the emergency setting—outpatient or emergency medicine. Its analgesic and sedative effects are used in premedication as part of balanced anaesthesia. It can also be used for anaesthesia or deep sedation for non-inducing treatments and procedures. The antagonistic effects on the µ-receptor can be used to reverse the side effects of opioids used both systemically and epidurally—pruritus, urinary retention, or respiratory depression—without affecting their analgesic effect [4,5,6,7]. The high efficacy of nalbuphine in pain relief has also been repeatedly confirmed accompanying veno-occlusive breakthroughs in patients with Sickle Cell Disease [8,9,10,11] and in neonates [12,13,14,15,16].

Nalbuphine provides fairly strong analgesia and a marked sedative effect, particularly expressed in the paediatric population. Agonistic influence on the ĸ receptor and its mediated interaction through G-protein and β-arestin 2 are responsible for a lower incidence of opioid-specific side effects such as nausea or pruritus [17] The antagonistic effect on the µ-receptor results in the reduction of analgesic effect of nalbuphine in the form of “ceiling effect”. Once the maximum possible level of analgesia has been reached, further dose escalation does not increase the analgesic potency. This is the primary reason why nalbuphine is placed among the medium potency opioid drugs [18]. 

Nalbuphine can be administered intravenously, intramuscularly, and subcutaneously. After oral administration, its bioavailability is very poor and is estimated to be around 12% in children and 11.8–44% in adult patients [19,20,21]. The parenteral-only route of intake significantly limits the use of nalbuphine in children, almost exclusively for inpatient and outpatient therapy. The side effects of nalbuphine such as nausea, vomiting, urinary retention, and pruritus are estimated to occur significantly less frequently with nalbuphine than with morphine [22,23,24]. 

The aim of this study was to analyse the comparison of side effects and the therapeutic efficacy of morphine and nalbuphine in pain management in children with cancer. 

## 2. Materials and Methods

### 2.1. Study Design

A prospective single-centre observational study analysed medical records of paediatric patients treated at the Clinic of Paediatric Haematology and Oncology of the University Hospital in Wroclaw (Poland), who developed mucositis during treatment. The study was conducted over two years between March 2019 and March 2020. The study followed the STROBE (Strengthening the Reporting of Observational Studies in Epidemiology) guidelines. 

### 2.2. Study Population 

The study included a group of 96 children with cancer during primary disease treatment. Children with severe pain (VAS > 4) caused by inflammatory changes of the gastrointestinal mucosa of the mucosal type, which is a side effect of the primary disease therapy, were included in the study. This level of pain in children requires the use of opioid analgesic (morphine or nalbuphine) in therapy. Children were included in the study at the time of treatment with opioids. Individual opioid selection, dosage, and treatment of side effects were applied according to the therapeutic regimens adopted in the Department of Haematology and Oncology. The analysis focused on comparing the analgesic efficacy, incidence of adverse effects, and withdrawal syndrome associated with the use of both opioids. The safety and efficacy of nalbuphine in children treated for cancer was also evaluated.

### 2.3. Pain Assessment

Pain levels were monitored regularly, twice daily, using age-appropriate pain rating scales: the Visual Analogue Scale (VAS), the Face, Legs, Activity, Cry, Consolability (FLACC), and the Wong–Baker Faces Pain Rating Scale (WB). The incidence of opioid side effects such as nausea and vomiting, constipation, difficulty urinating, pruritus, and the occurrence of post-treatment withdrawal syndrome were assessed daily during the study.

### 2.4. Ethical Considerations 

This study was approved by the independent Bioethics Committee of the Wroclaw Medical University (decision no. 517/2018). The study was carried out in accordance with the tenets of the Declaration of Helsinki and recommendations of good clinical practice. Strengthening the Reporting of Observational Studies in Epidemiology (STROBE) guidelines were followed in reporting. Written consent to the child’s participation in the study was obtained from every child’s parents or legal guardians. 

### 2.5. Statistical Analysis

The statistical analysis was performed using Statistica 13 software (TIBCO, Inc., Palo Alto, CA, USA). Arithmetic means, medians, standard deviations, quartiles, and range of variation (extreme values) were calculated for measurable variables. In the case of qualitative variables, their frequency was calculated (%). All studied quantitative variables were verified with the Shapiro-Wilk test used for determination of a distribution type. Comparisons of qualitative variables between groups were made using the Chi-squared test. Comparison of quantitative type results between groups was performed using the Mann-Whitney U test or the Kruskal-Wallis test. A comparative, within-group analysis between results obtained on consecutive days of therapy was performed using the Friedman test. The Spearman rank–order correlation analysis between selected variables was also conducted. An α = 0.05 was used for all comparisons.

## 3. Results

The study examined 96 cases of opioid drugs (nalbuphine or morphine) used in the treatment of children. Among the children receiving nalbuphine therapy, 30.77% (*n* = 20) of children were aged up to 4 years, 40.00% (*n* = 26) of young patients were aged 4 to 12 years, and 29.23% (*n* = 19) of them were aged over 12 years. In the group of children receiving morphine, 19.35% (*n* = 6) of children were aged up to 4 years, 45.17% (*n* = 14) were aged 4 to 12 years, and 35.48% (*n* = 11) of children were aged over 12 years. There were no statistically significant differences between the groups in terms of age. As many as 29.23% (*n* = 19) of those receiving nalbuphine therapy were doing so after a bone marrow transplant procedure. In the morphine-treated group, 35.48% (*n* = 11) of children were receiving the opioid after a bone marrow transplant procedure. There were no statistically significant differences in terms of age between the two groups of children after bone marrow transplantation. No significant differences were observed in the drug choice (*p* > 0.05) between the two groups.

### 3.1. Duration of Use for Nalbuphine and Morphine

Table 1 shows a comparison of the therapy duration depending on the drug received. For nalbuphine, the average therapy duration was 9.1 days (min–max: 2.0–31.0 days; SD = 5.8 days) and for morphine, the average therapy duration was 11.3 days (min–max: 2.0–31.0 days; SD = 7.0 days). The therapy duration was not statistically significantly different between the two groups analysed (*p* > 0.05).

### 3.2. Nalbuphine and Morphine Dosage

Table 2 shows the dosage of nalbuphine and morphine administered on consecutive days by continuous intravenous infusion.

### 3.3. Efficacy of Pain Relief 

**Figure 1:**
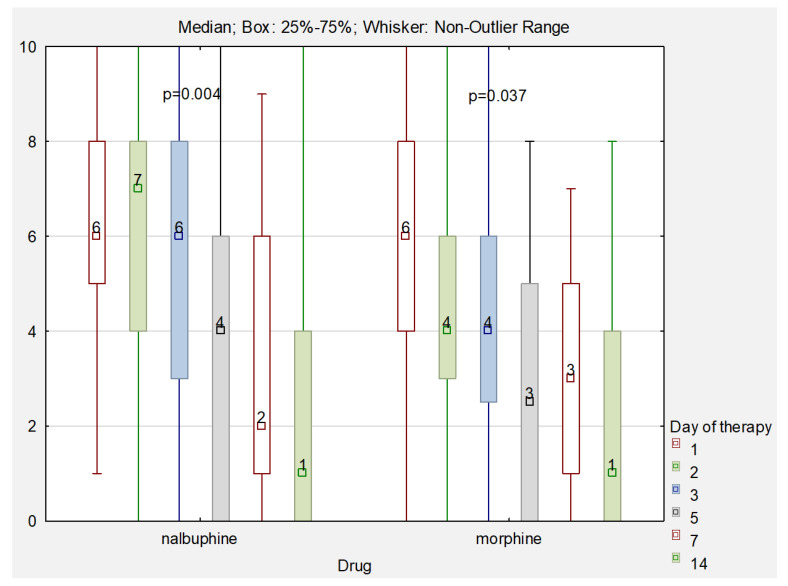
Comparison of pain levels on consecutive days of therapy.

Figure 1 shows a comparison of pain intensity scores obtained using a 10-point pain rating scale in patients receiving nalbuphine or morphine. Statistically significant differences (*p* < 0.05) were observed on the second day of therapy. The mean pain score for patients receiving nalbuphine was 6.22 (min–max: 1.0–10.0; SD = 2.4), and for those treated with morphine the mean score was 4.38 (min–max: 0.0–10.0; SD = 2.9). This indicates that on the second day of treatment, children receiving morphine experienced less pain compared to those receiving nalbuphine. No statistically significant differences in pain intensity were observed between children receiving both drugs on other days of therapy. In both cases, a statistically significant (*p* < 0.05) decrease in pain was observed on subsequent days of therapy.

### 3.4. Complications Associated with the Use of Opioid Drugs

#### 3.4.1. Nausea and Vomiting

There were no statistically significant differences in the incidence of nausea and vomiting on consecutive days of therapy between the groups of children receiving nalbuphine and morphine. (*p* > 0.05) (Figures S1 and S2—Supplementary).

#### 3.4.2. Constipation

On the first, third, and last day of therapy, the incidence of constipation in children receiving morphine was statistically significantly higher compared with those receiving nalbuphine (*p* < 0.05) (Table 3).

#### 3.4.3. Drowsiness

During the second and third days of treatment, there was a statistically significantly higher (*p* < 0.05) incidence of drowsiness in children receiving nalbuphine compared with those receiving morphine (Figure S3—Supplementary).

#### 3.4.4. Urinary Disturbances

There were no statistically significant differences in the incidence of urinary disturbances between the two patient groups compared on consecutive days of therapy except the last day. On that day, urinary problems occurred in 3.13% (*n* = 2) of patients receiving nalbuphine and in 19.35% (*n* = 6) of children receiving morphine. The difference was statistically significant (*p* < 0.05) (Table 4).

#### 3.4.5. Pruritus

Statistically significant differences were found in the incidence of pruritus between the two analysed groups (*p* < 0.05) on days 1, 2, 3, 5, and 7 (Table 5).

#### 3.4.6. Dysphoric Disorders, Agitation

The incidence of child behavioural disturbances manifested as agitation or dysphoria did not differ significantly over the course of treatment between the two study groups. The exception was the fifth day of therapy, when dysphoria was noted in 3 patients (12%) receiving morphine and did not occur at all in the nalbuphine treatment group (Figure S4—Supplementary).

#### 3.4.7. Onset of Withdrawal Syndrome (Abstinence)

Withdrawal syndrome occurred in 9.23% (*n* = 6) of patients receiving nalbuphine, and in 38.71% (*n* = 12) of patients receiving morphine. The difference was statistically significant (*p* < 0.05) (Table 6).

#### 3.4.8. Drug Change from Nalbuphine to Morphine

In 20% (*n* = 13) of patients treated with nalbuphine, a drug change to morphine was required. Such a change was made in five patients aged less than 4 years, and in eight patients aged between 10 and 15 years. In nine cases the reason for changing the drug was inadequate analgesia, while in four cases (1 young child and three adolescents—6% of children treated with nalbuphine) the reasons were anxiety, malaise, and irritability reported by the child.

## 4. Discussion

The management of pain in children is based on the 2012 WHO guidelines outlining the most important aspects of pain management in that age group of patients. Specific findings based on well-documented clinical trials are unfortunately not available. N. Schechter points out that the paediatric population is highly neglected in terms of potential use opioids for pain therapeutic opportunities [25]. New preparations are not tested in this group, and pharmaceutical companies are not interested in developing special preparations for this population group, so most of the regimens used are adapted, with some modifications, directly from the regimens for the treatment of adult patients. In 2019, Eccleston and associates analysed 704 reports of chronic pain management (neoplastic and non-neoplastic) in children. They did not find a single randomised study evaluating pain management methods in this age group. In their analysis, they rated the quality of all scientific data as very poor [26]. Although the bibliography contains some information and data regarding pain management pathways for children in palliative care, as well as certain recommendations on how to ensure their comfort, there is a large gap in the literature in the area of perioperative therapy of chronic diseases, especially neoplastic ones. In 2004, when analysing the problem of pain experienced by children during the treatment of neoplastic disease, Zernikow et al. pointed out that as many as 54% of suffering children indicate the direct consequences of antineoplastic treatment as the main reason for their pain, while medical procedures are claimed as the main cause of pain [27] by 42% of children suffering from severe pain.

The present study analysed the use of nalbuphine in children who developed mucositis during neoplastic treatment—a painful and impairing consequence of chemotherapy. Inflammatory changes of varying severity usually appear on days 3 to 10 after a cycle of chemotherapy and persist for up to about three weeks, with maximum discomfort experienced in the first two weeks [28]. Mucositis-type changes can vary in severity and impair a patient’s functioning in different ways. The study by Zernikov et al. showed that up to 88% of patients suffering from mucositis described the accompanying pain as severe or very severe [28].

In the present study, children who developed mucositis rated their pain as higher than 4 on a 10-point scale. Nalbuphine was administered to 65 children, i.e., 67.7% of all patients, while morphine was received by 31 children, i.e., 32.3% of all patients. In 20% of patients who received nalbuphine the drug was switched to morphine after several days, for various reasons. The change resulted from inadequate analgesic effect in 69% of cases, and from dysphoric disorder in 31% of cases. The statistical analysis of demographic data in children from both groups has not revealed any statistically significant differences. As for the duration of the use of both drugs, the shortest was 2 days and the longest was 31 days. The mean duration of nalbuphine use was 9.1 days, and for morphine, it was slightly longer―11.3 days. However, the difference in treatment duration between the two tested drugs was not statistically significant. 

Both nalbuphine and morphine were administered intravenously in single doses or by continuous infusion. The dosage of both drugs was determined individually and optimally for the patients. Nalbuphine given by continuous infusion was administered in a dose range of 0.07 to 0.16 mg/kg/h, and morphine was administered in a dose range of 0.03 to 0.14 mg/kg/h. The dose range showed a large scatter of individually administered doses, making it difficult to estimate an effective analgesic dose.

A comparison of the pain relief efficacy of the two drugs showed that both nalbuphine and morphine were effective in relieving pain. Only on the second day of treatment, the comparison of the efficacy of both drugs showed a significantly greater effectiveness of morphine (*p* < 0.05). The mean pain level in children treated with nalbuphine was 6.2, and in patients treated with morphine it was 4.4. On other days, no statistically significant differences in the level of pain were observed between the two groups of patients. 

The incidence of side effects during nalbuphine and morphine treatment was analysed in detail. The occurrence of side effects was considered extremely important because the side effects of opioid drugs can be particularly severe for children. The data published in the literature, and the theoretical rationale for nalbuphine, reveal that the incidence of complications during its use should be much lower than in case of morphine. In 2015, Zheng et al. analysed 15 clinical trials that compared the potency and incidence of adverse reactions for nalbuphine and morphine. They found no significant differences in the levels of pain relief; however, they observed a significantly lower incidence of complications such as pruritus, nausea, and vomiting, and episodes of respiratory depression when nalbuphine was used [22]. Unfortunately, all the studies they referred to concerned the administration of both drugs in adult patients and in short-term treatment involving postoperative pain management. All the papers on the use of nalbuphine for pain relief in children—Krishnan [29], Bressolle [14], Schutz-Machata [19] and Larsen [30]—analyse its use in perioperative pain management. All of these publications report that nalbuphine is less likely to cause opioid-specific side effects. However, no work addressing the aspect of long-term use of nalbuphine in children was found in the literature.

In the analysed material, nausea occurred in 19.3% of children receiving morphine on the first day of treatment, and only in 9.2% of children receiving nalbuphine. A slightly higher proportion of patients receiving morphine reported nausea throughout their treatment. Statistical analysis showed no statistically significant differences (*p* > 0.05) in the incidence of this adverse event between the compared drugs, but it should be remembered that in children receiving chemotherapy, anti-emetic prophylaxis is an integral component of the therapy. Most of the children in the study received antiemetics as part of the treatment protocol, which greatly influences the assessment of the frequency of this side effect.

The situation was similar in case of the incidence of vomiting. Again, the statistical analysis showed no statistically significant differences in the incidence of vomiting between the two groups of children. Similar to the case of the analysed incidence of nausea, the reasons for this state of affairs may be attributed to the too-small number of the studied groups and use of antiemetics in children in a regular way, as an element of routine therapy accompanying chemotherapy.

Assessing the incidence of constipation as an opioid side effect is quite different. Based on its mechanism of action, it could be expected that nalbuphine, as a µ-receptor antagonist, would contribute much less to constipation. It should be emphasized that constipation and its treatment are highly discomforting and can be very unpleasant especially for a child suffering from mucositis. The results of our study indicate that the problem of constipation occurred significantly less frequently in children treated with nalbuphine. In the group of children receiving morphine, a higher incidence of constipation persisted throughout treatment. On the last day of opioid treatment, constipation occurred in 12.9% of patients receiving morphine and only 1.6% receiving nalbuphine.

Urination problems are another side effect of opioids. Micturition disorders during opioid use are due to their effects on parasympathetic innervation of the bladder through µ- and 𝛿-opioid receptors, but not through the ĸ-opioid receptor; therefore, the unique receptor activity of nalbuphine does not cause micturition disorders in patients treated with nalbuphine. In addition, there is a documented literature showing the efficacy of nalbuphine in treating µ-agonist opioid-induced urinary retention without compromising the quality of analgesia [23,24,31,32].

The present study compared the incidence of micturition disorders in children treated with morphine and nalbuphine. We found significantly more frequent micturition problems in children receiving morphine than nalbuphine. Moreover, these disorders persisted throughout morphine therapy, while with nalbuphine the problem of urination in children becomes insignificant. This is especially important because effective treatment of severe micturition disorders in children in the clinical setting is limited to bladder catheterization, which is a painful, traumatising procedure for children and risks other complications like infection.

Pruritus is another common side effect of opioids. This unpleasant sensation may affect not only the superficial layers of the skin, but also the mucosa and conjunctivae. There is a very close relationship between pruritus and pain. Although these are separate stimuli with separate conduction pathways, they are experienced by patients as related sensations; both can be equally distressing to the patient, and both can affect each other. 

Nalbuphine is much less likely to cause pruritus than morphine. Zheng estimates that pruritus occurs in 20.5% of cases when morphine is used in the perioperative period, whereas it occurs in only 4.7% of patients during nalbuphine treatment [22]. Nalbuphine has sometimes been used with great success to relieve pruritus after opioid use. Tubog et al. reviewed 17 studies that evaluated the efficacy of nalbuphine in preventing pruritus associated with the epidural administration of morphine, fentanyl, sufentanil, and hydromorphone. Their analysis revealed a high efficacy of the preventive use of nalbuphine in relieving pruritus, without affecting the quality of analgesia [33]. R. Januzzi, on the basis of analysed studies, believes that nalbuphine should be a priority drug in treating opioid-induced pruritus, especially in epidural opioid administration [34]. In our study, pruritus was found to occur much less frequently in children receiving nalbuphine than morphine. In the morphine group, 29% of patients complained of pruritus on the first day, while in the group of children receiving nalbuphine, this occurred in 6.1% of patients. Similar proportions of pruritus persisted throughout the treatment period, with a significantly higher incidence in the morphine group.

Our study is consistent with previously cited work on the incidence of side effects [14,19,22]. Nalbuphine is less likely to cause unpleasant side effects for children such as nausea, vomiting, urinary retention, constipation, and itching than morphine, which not only improves the child’s comfort but reduces the need for additional medications and medical procedures.

Patients receiving opioid drugs often complain of excessive drowsiness. However, for children treated in an inpatient unit, sleepiness was perceived as a limiting factor neither by children nor by their caregivers. On the contrary, drowsiness, especially in the early stages of treatment, was usually seen by caregivers as a positive aspect of the treatment. Bressolle estimates the incidence of drowsiness in children during the use of nalbuphine at 32% [13], and an analysis of the material obtained by the present authors yields results corresponding with those obtained in the cited study. On the first day of opioid treatment, excessive sleepiness occurred in 38% of children who received nalbuphine, and 19.3% of those who received morphine. The higher incidence of drowsiness in children treated with nalbuphine persisted during the first days of therapy, with the incidence of drowsiness similar in both groups as time progressed. However, the incidence of drowsiness in children receiving nalbuphine decreased significantly over time, indicating a gradual development of tolerance to the effect.

The occurrence of dysphoric disorders was the adverse effect of opioid drugs that was of greater concern for children and their caregivers than drowsiness. The disorders manifested in different forms: excessive anxiety, agitation, and unusual behaviour. In one case, auditory hallucinations were reported. Behavioural disturbance tends to be dominated by dysphoric disorders, which are not altogether pleasant for patients and were assessed negatively as an emotional state rather than as euphoria, whose positive emotional consequences might consequently induce the patient to abuse the drug [35]. In the abovementioned study, on the first day of opioid use, behavioural disturbances were observed in 10% of children receiving morphine and in 10.8% of those receiving nalbuphine. The proportion of dysphoric disorders remained similar over the course of the treatment, with no significant statistical differences. The exception was on the fifth day of the treatment, when behavioural disturbances were reported by three patients receiving morphine (12%) and none of those receiving nalbuphine. It is also worth noting that a comparison of the incidence of reported dysphoric episodes at the beginning and end of the treatment indicates that for morphine, the incidence of behavioural disturbances in children increases with the increasing duration of treatment. On the first day, dysphoric episodes were reported in 10% (*n* = 3) of children receiving morphine, and on the last day of treatment, the incidence was 32.3% (*n* = 10) in patients receiving the medication. No similar correlation was found for nalbuphine.

When both nalbuphine and morphine were used, there was not a single case of respiratory depression.

Another important aspect of the use of opioid drugs is the occurrence of withdrawal symptoms after the end of treatment. They involve somatic disorders such as tremors, tachycardia, excessive sweating, food intolerance, abdominal pain, vomiting, diarrhoea, and psychiatric disorders: anxiety, malaise, insomnia, disorientation, or behavioural disturbances. Opioid dependence leading to withdrawal syndrome can be expected as early as after 5 days of drug use, although such symptoms are most commonly found in children treated for longer than 7 days. However, in children who have received opioids for more than 14 days, the development of such dependence can be expected with a high degree of certainty [36]. This study found a significantly higher incidence of withdrawal syndrome in patients receiving morphine. It developed in as many as 38.7% of all children treated with this drug. Among patients treated with nalbuphine, symptoms of withdrawal were present in 9.2% of children after the end of treatment. Neither morphine nor nalbuphine showed a statistically significant correlation between the occurrence of withdrawal syndrome and the duration of the treatment, which seems surprising in light of both the literature data and clinical practice. This may be due to the considerable dispersion in the duration of the use of both drugs (from 2 to 31 days), and the relatively small group of patients who received the drugs for more than two weeks. In the group of 65 children treated with nalbuphine, 28 received the drug for more than 10 days, 14 were treated for more than 14 days, and 3 patients were treated for more than 20 days. There were 15 children treated with morphine for more than 10 days, with 5 receiving it for more than 14 days, and 5 for more than 20 days.

### Study Limitation

This study has some limitations. Firstly, it was not a randomised study because of the great difficulty in designing a randomised trial of pain treatment in this group of patients. It was a single-centre observational study based on the therapeutic regimens used in the Department of Paediatric Haematology and Oncology. The wide range of drug doses and the wide age range of patients posed an additional difficulty in assessing the efficacy of analgesia. Another limitation was related to the relatively small number of patients. The reason for this was that the COVID-19 pandemic significantly limited the possibility of continuing the study in the Department of Paediatric Haematology and Oncology. The focus was therefore on assessing the safety of nalbuphine over a longer period of time and comparing the incidence of side effects of both drugs.

## 5. Conclusions

Nalbuphine is a drug that can be successfully used in children during cancer treatment for a period of several days or even weeks. Compared to morphine, the side effects typical of opioid use are less common, and the incidence decreases over time. The development of tolerance requiring dose escalation was not observed during nalbuphine use and features of post-therapy abstinence syndrome were much less frequent than in morphine use. This is the first study to evaluate this aspect of long-term nalbuphine use and the results encourage further research in this direction.

## Figures and Tables

**Table 1:** A Comparison of therapy period depending on the drug used.

	Drug														*p*-Value *
	Nalbuphine							Morphine							
	$\bar{x}$	Me	Min	Max	Q1	Q3	SD	$\bar{x}$	Me	Min	Max	Q1	Q3	SD	
**Time [Days]**	9.1	7.0	2.0	31.0	5.0	13.0	5.8	11.3	9.0	2.0	31.0	6.0	15.0	7.0	0.13

$\bar{x}$ —mean; Me—median; Min—minimum value; Max—maximum value; Q1—lower quartile; Q3—upper quartile; SD—standard deviation * Mann-Whitney U-test

**Table 2:** Comparing nalbuphine and morphine dosing by continuous intravenous infusion on consecutive days of therapy.

Dose [mg/kg/h]	Nalbuphine							Morphine						
	$\bar{x}$	Me	Min	Max	Q1	Q3	SD	$\bar{x}$	Me	Min	Max	Q1	Q3	SD
Day 1	0.07	0.06	0.02	0.13	0.05	0.09	0.03	0.03	0.03	0.00	0.14	0.02	0.04	0.02
Day 2	0.08	0.08	0.03	0.14	0.05	0.10	0.03	0.04	0.03	0.01	0.14	0.03	0.04	0.02
Day 3	0.08	0.07	0.02	0.14	0.06	0.10	0.03	0.04	0.04	0.01	0.14	0.03	0.04	0.02
Day 5	0.07	0.07	0.01	0.15	0.05	0.09	0.03	0.04	0.04	0.01	0.14	0.03	0.05	0.02
Day 7	0.08	0.08	0.02	0.15	0.05	0.10	0.04	0.03	0.03	0.01	0.14	0.02	0.04	0.03
Day 10	0.07	0.06	0.01	0.15	0.03	0.08	0.04	0.04	0.04	0.02	0.14	0.02	0.04	0.03
Day 14	0.07	0.07	0.02	0.16	0.03	0.10	0.05	0.04	0.02	0.02	0.14	0.02	0.03	0.04
*p*-value				0.73						0.38				

**Table 3:** Comparison of the incidence of constipation between patients receiving nalbuphine and morphine.

Gastrointestinal Disorders (Constipation)		Drug				*p*-Value *
		Nalbuphine		Morphine		
		*n*	%	*n*	%	
Day 1	No	61	93.85	24	77.42	0.018
	Yes	4	6.15	7	22.58	
Day 2	No	57	91.94	23	79.31	0.085
	Yes	5	8.06	6	20.69	
Day 3	No	51	89.47	20	71.43	0.035
	Yes	6	10.53	8	28.57	
Day 5	No	40	90.91	21	80.77	0.221
	Yes	4	9.09	5	19.23	
Day 7	No	26	83.87	15	78.95	0.660
	Yes	5	16.13	4	21.05	
Day 10	No	20	95.24	12	92.31	0.724
	Yes	1	4.76	1	7.69	
Day 14	No	12	100.00	8	100.00	-
	Yes	0	0.00	0	0.00	
Final day	No	63	98.44	27	87.10	0.020
	Yes	1	1.56	4	12.90	

*n*—number of persons, %—percentage; * Chi-squared test.

**Table 4:** Comparison of the incidence of urinary disturbances between patients receiving nalbuphine or morphine.

Inability to Urinate, Bladder Catheterisation		Drug				*p*-Value *
		Nalbuphine		Morphine		
		*n*	%	*n*	%	
Day 1	No	58	90.63	26	83.87	0.335
	Yes	6	9.38	5	16.13	
Day 2	No	53	85.48	25	86.21	0.927
	Yes	9	14.52	4	13.79	
Day 3	No	49	85.96	25	86.21	0.976
	Yes	8	14.04	4	13.79	
Day 5	No	42	95.45	22	84.62	0.118
	Yes	2	4.55	4	15.38	
Day 7	No	29	93.55	15	78.95	0.123
	Yes	2	6.45	4	21.05	
Day 10	No	20	95.24	10	76.92	0.107
	Yes	1	4.76	3	23.08	
Day 14	No	12	100.00	6	75.00	0.068
	Yes	0	0.00	2	25.00	
	Yes	6	9.38	5	16.13	
Final day	No	62	96.88	25	80.65	0.008
	Yes	2	3.13	6	19.35	

*n*—number of persons, %—percentage; * Chi-squared test.

**Table 5:** Comparison of the incidence of pruritus between patients receiving nalbuphine or morphine.

	Pruritus	Drug				*p*-Value *
		Nalbuphine		Morphine		
		*n*	%	*n*	%	
Day 1	No	61	93.85	22	70.97	0.002
	Yes	4	6.15	9	29.03	
Day 2	No	57	91.94	19	65.52	0.002
	Yes	5	8.06	10	34.48	
Day 3	No	52	91.23	18	62.07	0.001
	Yes	5	8.77	11	37.93	
Day 5	No	43	97.73	17	65.38	<0.001
	Yes	1	2.27	9	34.62	
Day 7	No	29	93.55	12	66.67	0.014
	Yes	2	6.45	6	33.33	
Day 10	No	18	85.71	11	84.62	0.930
	Yes	3	14.29	2	15.38	
Day 14	No	10	83.33	8	100.00	0.224
	Yes	2	16.67	0	0.00	
*p*-value		0.638		0.125		

n—number of persons, %—percentage; * Chi-squared test.

**Table 6:** Comparison of the incidence of withdrawal syndrome depending on the drug used.

		Withdrawal Syndrome				*p*-Value *
		No		Yes		
		*n*	%	*n*	%	
Drug	Nalbuphine	59	90.77	6	9.23	<0.001
	Morphine	19	61.29	12	38.71	

*n*—number of persons, %—percentage; * Chi-squared test.

## Data Availability

The data may be obtained by contacting the corresponding author.

## Supplementary Materials

The following are available online at https://www.mdpi.com/article/10.3390/cancers14153617/s1, Figure S1: Incidence of nausea in patients receiving nalbuphine or morphine; Figure S2: Incidence of vomiting in patients receiving nalbuphine or morphine; Figure S3: Incidence of drowsiness in patients receiving nalbuphine or morphine; Figure S4: Incidence of behavioural disturbances in patients receiving nalbuphine or morphine.
