# Supplementary material for: Comparison of Side Effects of Nalbuphine and Morphine in the Treatment of Pain in children with Cancer: A Prospective Study
Source: Cancers (Basel). 2022 Jul 25;14(15):3617. doi: 10.3390/cancers14153617 (PMC9330467; doi:10.3390/cancers14153617)
Supplement: Supplementary file 1 [file cancers-14-03617-s001.zip › cancers-1832790-supplementary.pdf]

cancers

Article

Supplementary Materials: Comparison of side effects of nalbuphine and morphine in the treatment of pain in children with cancer: a prospective study

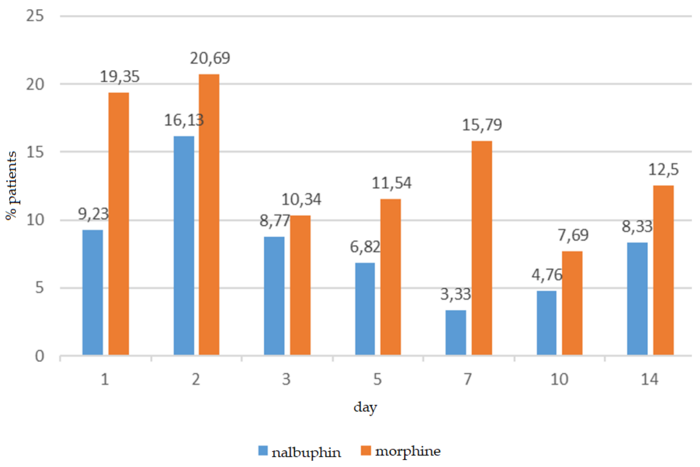

Figure S1. Incidence of nausea in patients receiving nalbuphine or morphine.

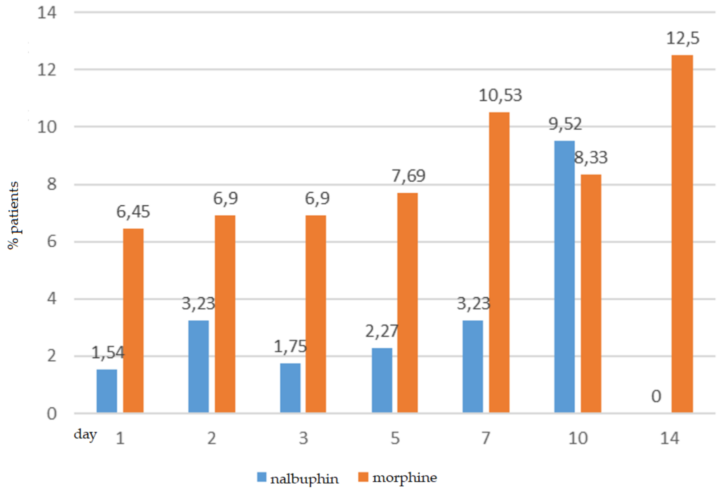

Figure S2. Incidence of vomiting in patients receiving nalbuphine or morphine.

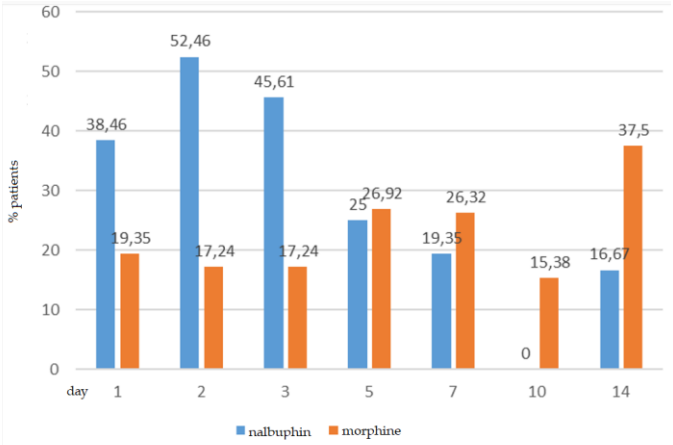

Figure S3. Incidence of drowsiness in patients receiving nalbuphine or morphine.

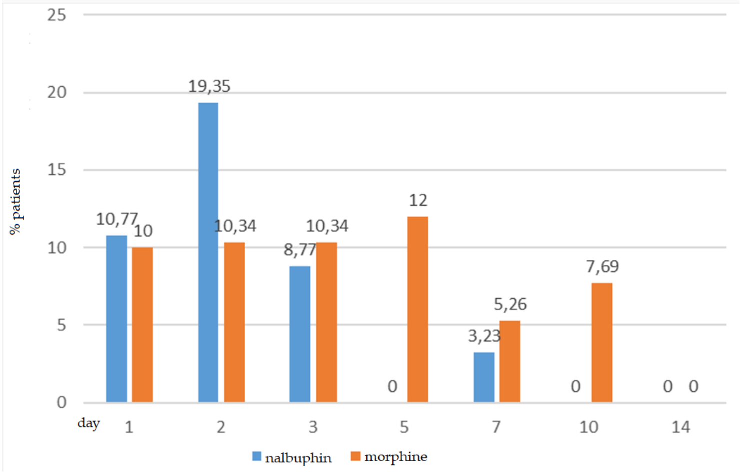

Figure S4. Incidence of behavioural disturbances in patients receiving nalbuphine or morphine.
